# Coexistence of Endodermal Cyst and Neurofibromatosis Type 1: A Case Report

**DOI:** 10.7759/cureus.104336

**Published:** 2026-02-26

**Authors:** Kouichi Takano, Yasuo Inoue, Yuta Oi, Takaharu Kawajiri, Takashi Hohri

**Affiliations:** 1 Department of Neurosurgery, National Hospital Organization, Maizuru Medical Center, Kyoto, JPN

**Keywords:** benign cystic lesion, endodermal cyst, extra-axial lesion, neurofibromatosis type 1, posterior fossa

## Abstract

Endodermal cysts (ECs) are rare benign cystic lesions of the central nervous system. Neurofibromatosis type 1 (NF1) is a genetic disorder associated with neural tumors, but co-occurrence with EC is exceedingly uncommon. We report the case of a 29-year-old male with NF1, characterized by café-au-lait spots and a family history, who presented with headache and left upper limb deficits following minor head trauma. Imaging revealed a posterior fossa tumor. Due to progressive neurological decline, the mass was surgically resected. Intraoperative findings showed a yellowish-white, jelly-like accumulation. Pathological examination confirmed the diagnosis of EC, which was characterized by a wall lined with ciliated epithelium, but lacking skin appendages in the cyst wall. This case suggests that ECs should be included in the differential diagnosis of extra-axial tumors in the posterior fossa. Given the rarity of ECs, a potential association with NF1 is suspected in this case, although the causal relationship remains unclear.

## Introduction

Endodermal cysts (ECs) are rare benign cystic lesions composed of endoderm-derived cells that possess cilia and mucus-secreting cells and constitute 0.01-0.35% of all central nervous system tumors. Embryologically, ECs are thought to arise from the abnormal separation of the endoderm and notochord during early development. These lesions are typically located in the intradural extramedullary space and can present with compressive neurological symptoms. On imaging, they often appear as well-circumscribed cystic masses with signal intensities varying according to their protein content [1-4]. This report presents a case of EC in a patient who was newly diagnosed with neurofibromatosis type 1 (NF1). Various neural tumors are known to be associated with NF1, such as schwannomas. Cases of arachnoid cysts have been reported as complications of benign cystic diseases, but we were unable to identify any such cases with ECs [5-7]. Therefore, we report this case as the first example of a possible association of EC with NF1. Clarifying the differential diagnosis for lesions around the brainstem is crucial for surgical planning. Identifying the possible coexistence of EC and NF1 may facilitate more precise strategies and improved management of these rare presentations.

## Case presentation

A 29-year-old Japanese male presented with headache, neck pain, and numbness in his left hand following a minor head injury. His Glasgow Coma Scale was E4V5M6, with muscle weakness and sensory deficits in the left upper limb. Café-au-lait spots were found on the trunk, and the patient had a family history of NF1. Diagnosis of NF1 was made clinically using the NIH diagnostic criteria, based on the café-au-lait spots and family history in a first-degree relative. Genetic testing was not performed [8].

**Figure 1:**
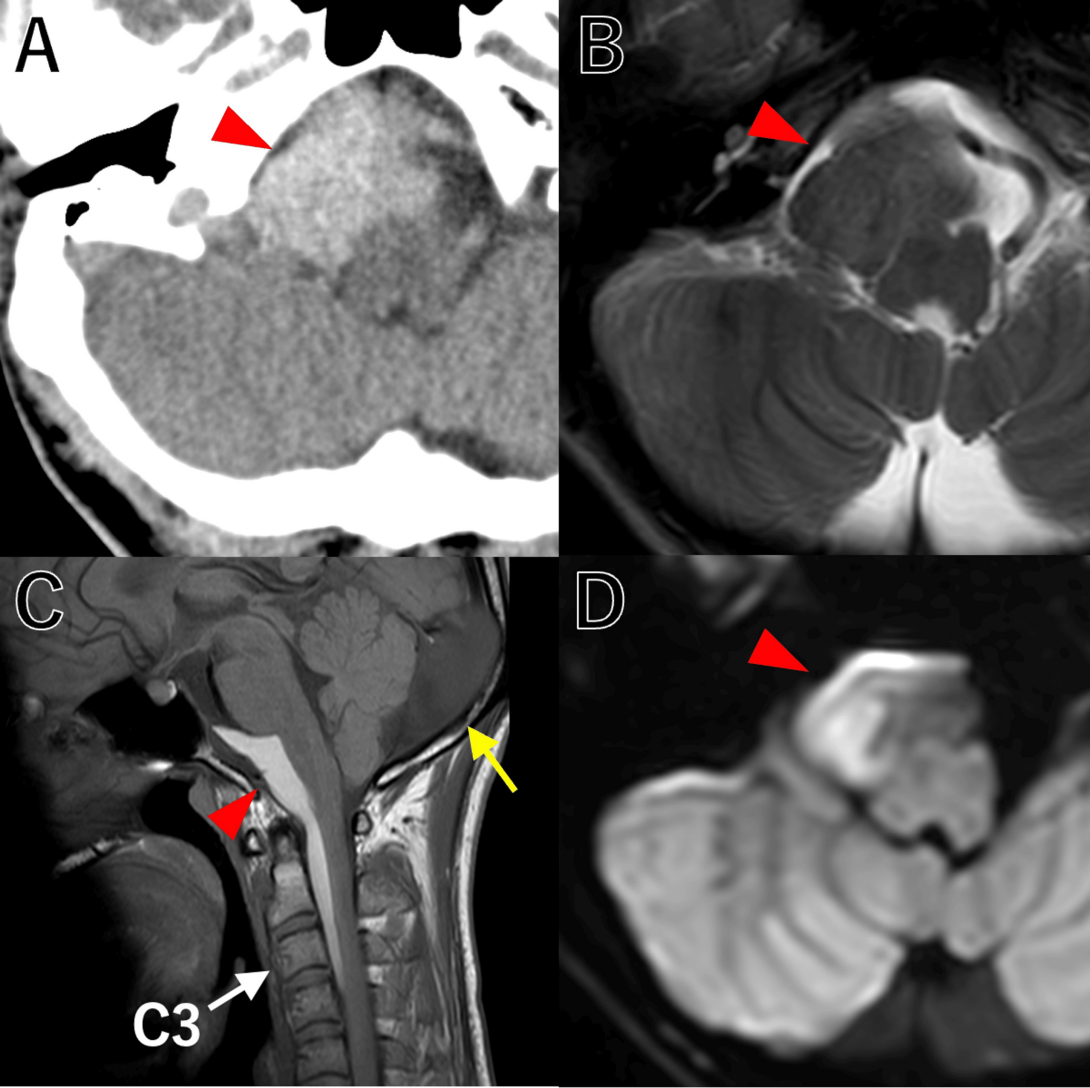
CT and MRI findings. Plain CT showed a high-density lesion (red arrowhead) in the right lateral medullary cistern adjacent to the jugular foramen (A). MRI showed a lesion (red arrowhead) with a T2 isointense signal (B), a T1 hyperintense signal (C), and DWI mixed intensity (D) extending from the posterior cranial fossa. The tumor extended into the cervical spinal canal (White arrow: the C3 vertebral body). A subtentorial arachnoid cyst (yellow arrow) was also present.

Computed tomography (CT) and magnetic resonance imaging (MRI) revealed an extra-axial tumor of approximately 10 cm in craniocaudal size extending from the right cerebellopontine angle to the premedullary cistern and into the cervical spinal canal at the C3 level. The tumor showed hyperdensity on CT, an isointense signal on T2-weighted MRI, and a hyperintense signal on T1-weighted MRI. Diffusion-weighted images showed a mixture of high and low signal intensity. The patient also had a subtentorial arachnoid cyst (Figure 1).

**Figure 2:**
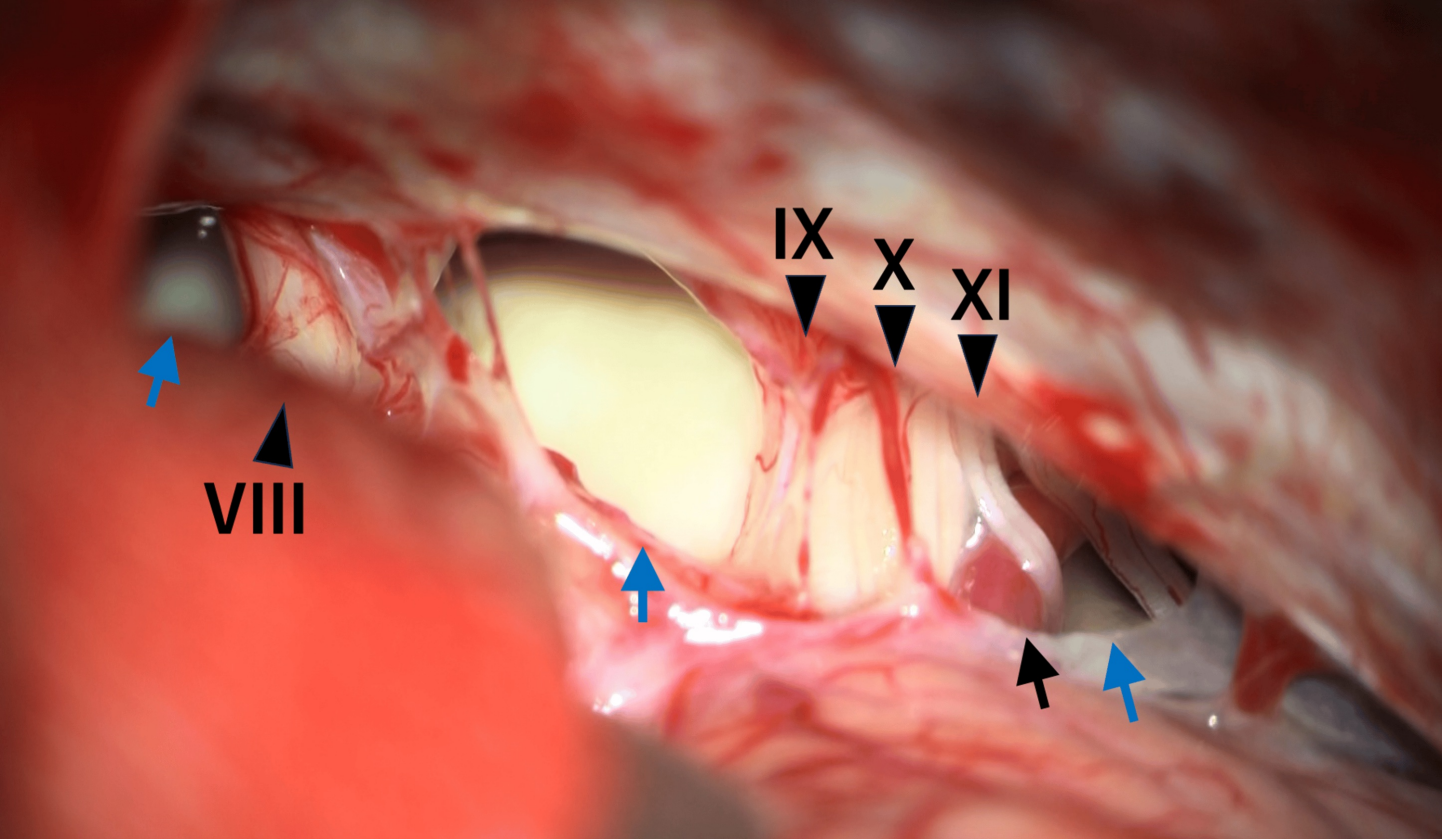
Intraoperative findings after right-sided lateral suboccipital craniotomy. A cream-colored cyst enclosed in a capsule was visualized (blue arrows). Cranial nerves (VIII: vestibulocochlear, IX: glossopharyngeal, X: vagus, XI: accessory) and the anterior inferior cerebellar artery (black arrow) are identified. The tumor extended beyond the lower cranial nerves to the caudal region.

Initially, a jugular foramen schwannoma associated with NF1 was suspected. The high density on CT suggested tumor hemorrhage. The patient presented with a two-week history of progressive neurological deterioration. Due to decreased consciousness, right 6th and 7th cranial nerve palsy, loss of light reflex, and onset of seizures, the patient underwent surgery via a right-sided lateral suboccipital craniotomy. The area from the cerebellopontine cistern to the lateral medullary cistern was filled with a yellowish-white, jelly-like accumulation covered by a membrane. No hematoma components were observed within the cyst. After complete removal of the contents, the abducens nerve was identified deep in the cyst. The membrane partly adhered to the brainstem surface and cranial nerves, and partial excision was performed (Figure 2). Intraoperative frozen section examination was not performed.

**Figure 3:**
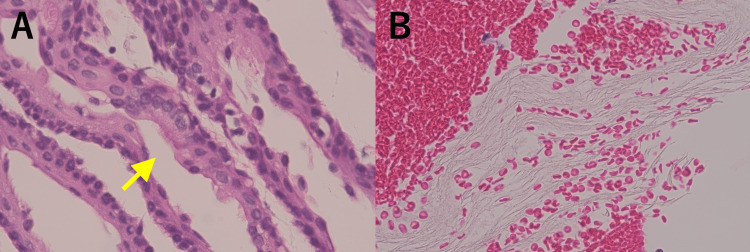
Histopathology. The cyst wall was lined with ciliated epithelium and stratified squamous epithelium (A). Cilia are evident (yellow arrow). The cyst contents consisted of layered, keratin-like material (B).

Pathological examination revealed that the cyst wall was composed of thin, loose fibrous connective tissue and that the lumen was lined with ciliated epithelium and stratified squamous epithelium. No skin appendages were observed. The cystic contents consisted of layered, keratin-like material, but no identifiable epithelial components (Figure 3). Immunohistochemical markers were not used in the histopathological diagnosis. Based on these findings, the lesion was diagnosed as EC.

**Figure 4:**
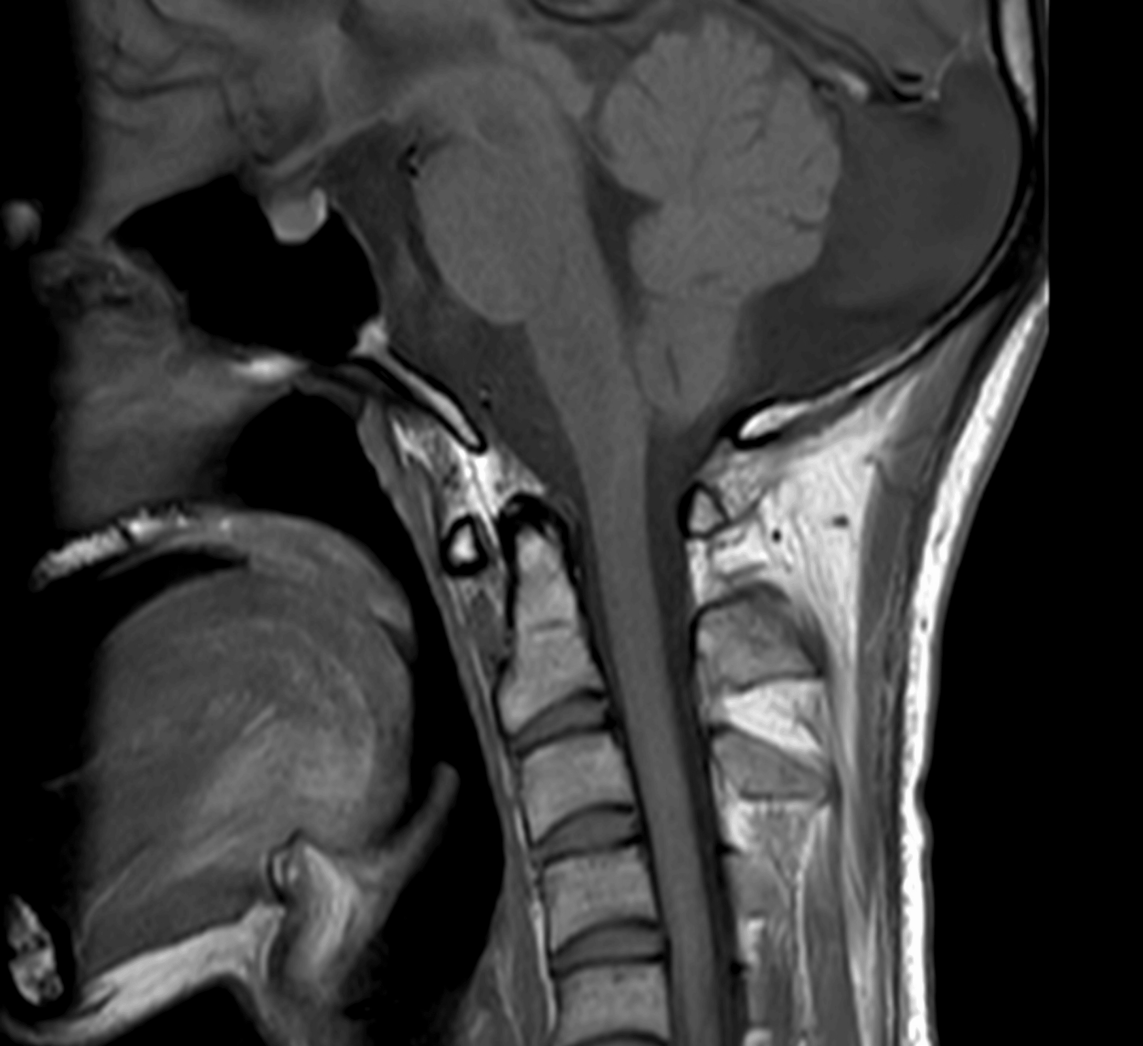
Postoperative follow-up image. T1-weighted sagittal images obtained two months postoperatively showed no evidence of residual tumor or recurrence.

The level of consciousness and 6th and 7th cranial nerve palsy gradually improved after surgery. No clinical or radiographic recurrence was observed during the 5-month follow-up period (Figure 4).

## Discussion

In this case, it was difficult to diagnose the lesion preoperatively. First, the localization of the lesion appeared continuous with the jugular foramen, which led to initial suspicion of a jugular foramen schwannoma associated with NF1. Schwannomas typically do not exhibit high density on CT, so in this case, including the rapid worsening of symptoms, we suspected hemorrhage in the tumor. Second, MRI findings in cystic lesions show varying signal intensities depending on the nature of the cyst fluid [1,9], which makes it difficult to diagnose cysts based solely on MRI signal intensity. The lesion was finally diagnosed as EC by pathological examination. While most ECs follow a slow course, some may progress rapidly due to intracystic hemorrhage or meningitis caused by leakage of cyst contents [1,10-12]. Intraoperative findings revealed no evidence of hemorrhage in the cyst. Therefore, it was inferred that the cyst wall ruptured due to head trauma, allowing inflammation to spread around the brainstem and produce diverse symptoms, including brainstem dysfunction.

ECs are benign cystic lesions composed of epithelium similar to that found in endoderm-derived tissues, such as airway epithelium and intestinal epithelium. ECs include lesions previously referred to as enterogenous cysts, neuroenteric cysts, bronchogenic cysts, or epithelial cysts [1], and occur mainly in the posterior cranial fossa and intradural-extramedullary space of the spinal canal [1,13]. Macroscopically, ECs appear as a thin-walled, white or yellow cyst containing a gelatinous or creamy fluid. Histopathologically, the cyst wall is lined by columnar epithelium with cilia and mucus, and may show squamous metaplasia. Differential diagnoses include dermoid cysts, which originate from ectodermal tissue and are characterized by inclusion of skin appendages (hair follicles, sebaceous glands, sweat glands) in the cyst wall; and epidermoid cysts, which develop from vestigial embryonic tissue and are lined by a thin stratified squamous epithelium with retention of keratinized material within. Thus, these cysts contain abundant keratin and exhibit a pearl-like luster macroscopically. In contrast, ECs originate from endodermal tissue and are characterized by the absence of ectodermal components such as skin appendages [14]. 

NF1 was first described in 1882 by the German physician Friedrich Daniel von Recklinghausen, and thus, is also known as von Recklinghausen disease [15,16]. NF1 can cause various tumors of the nervous system, including neurofibroma, schwannoma, and glioma. Several cases of NF1 coexisting with arachnoid cysts have been reported, but NF1 with EC has not been documented to date. Also, reports of NF1 and arachnoid cysts have not addressed their embryological causality [5-7]. While arachnoid cysts are relatively common and may coincidentally occur with NF1, ECs are extremely rare. This suggests that NF1 may be a contributing factor. Our case also had a subtentorial arachnoid cyst, emphasizing the potential for NF1 to coexist with benign cystic diseases, including rare cysts. This case is the first report of NF1 combined with EC. Accumulation of similar cases will help to determine if there is a causal relationship between NF1 and EC.

## Conclusions

We encountered a case of NF1 associated with EC. Although ECs are rare, they should be considered in the differential diagnosis of extra-axial lesions in the posterior fossa. There is a need for further examination of the relationship of NF1 with rare cystic diseases.
